# TREM Receptors Connecting Bowel Inflammation to Neurodegenerative Disorders

**DOI:** 10.3390/cells8101124

**Published:** 2019-09-21

**Authors:** Gianfranco Natale, Francesca Biagioni, Carla Letizia Busceti, Stefano Gambardella, Fiona Limanaqi, Francesco Fornai

**Affiliations:** 1 Department of Translational Research and New Technologies in Medicine and Surgery, University of Pisa, Via Roma 55, 56100 Pisa, Italy; gianfranco.natale@med.unipi.it (G.N.); f.limanaqi@studenti.unipi.it (F.L.); 2 I.R.C.C.S Neuromed, Via Atinense 18, 86077 Pozzilli, Italy; francesca.biagioni@neuromed.it (F.B.); carla.busceti@neuromed.it (C.L.B.); stefano.gambardella@neuromed.it (S.G.)

**Keywords:** dysbiosis, microbiome, inflammatory bowel disease, gut-brain-axis, neurodegeneration, TREM-1, TREM-2, myeloid-derived cells, autophagy

## Abstract

Alterations in Triggering Receptors Expressed on Myeloid cells (TREM-1/2) are bound to a variety of infectious, sterile inflammatory, and degenerative conditions, ranging from inflammatory bowel disease (IBD) to neurodegenerative disorders. TREMs are emerging as key players in pivotal mechanisms often concurring in IBD and neurodegeneration, namely microbiota dysbiosis, leaky gut, and inflammation. In conditions of dysbiosis, compounds released by intestinal bacteria activate TREMs on macrophages, leading to an exuberant pro-inflammatory reaction up to damage in the gut barrier. In turn, TREM-positive activated macrophages along with inflammatory mediators may reach the brain through the blood, glymphatic system, circumventricular organs, or the vagus nerve via the microbiota-gut-brain axis. This leads to a systemic inflammatory response which, in turn, impairs the blood-brain barrier, while promoting further TREM-dependent neuroinflammation and, ultimately, neural injury. Nonetheless, controversial results still exist on the role of TREM-2 compared with TREM-1, depending on disease specificity, stage, and degree of inflammation. Therefore, the present review aimed to provide an update on the role of TREMs in the pathophysiology of IBD and neurodegeneration. The evidence here discussed the highlights of the potential role of TREMs, especially TREM-1, in bridging inflammatory processes in intestinal and neurodegenerative disorders.

## 1. Introduction

The human microbiota is the community of microorganisms that commensally live in symbiosis on the skin and mucosa of digestive, respiratory, and genito-urinary tracts. The collective genomes of microorganisms residing in these environmental niches form the microbiome, which is also referred to as metagenome [1]. The gastrointestinal microbiota (GIM) includes the largest number of microbial species. The nature of GIM varies significantly based on a number of conditions, such as diet, inflammatory disease, and drug intake [2]. Among these, chronic inflammation has received special attention due to marked alteration in microbiota and the concomitant association with central nervous system (CNS) disorders.

Marked alteration of the GIM occurs during inflammatory bowel disease (IBD), which refers to a variety of chronic inflammatory conditions up to ulcerative colitis (UC) and Crohn’s disease (CD). Emerging evidence suggests that intestinal dysbiosis may worsen the development of chronic intestinal disorders. In this context, bacterial compounds are recognized by specific receptor complexes named Pattern Recognition Receptors (PRRs), which are exposed to the intestinal immune cell membranes. This is the case of Nucleotide-binding and oligomerization domain (NOD)-Like Receptors (NLRs) and Toll-Like Receptors (TLRs), which enhance inflammation upon recognition of Microbe-Associated Molecular Patterns (MAMPs) [3,4]. About two decades ago, a new class of receptors was identified, namely Triggering Receptors Expressed on Myeloid cells (TREMs). These receptors are expressed on the plasma membrane of myeloid-derived cells, including neutrophils, monocytes, macrophages, osteoclasts, and glial cells [5,6]. TREMs are implicated in a variety of biological functions, including inflammation and immunity, coagulation, bone metabolism, cell differentiation, neuroplasticity, and neurodevelopment. At present, three TREM members on chromosome 6p21 in humans (TREM-1 to 3), and four TREM-like transcripts (TREML1-4), previously named novel Ig-like receptors, have been identified [6]. TREMs are typical membrane proteins with three domains: 1) a single, ligand-binding extracellular immunoglobulin-like domain; 2) a transmembrane domain, which activates myeloid cells by interacting via oppositely-charged residues with the 12-kDa DNAX Activating Protein (DAP12, a trans-membrane component containing an Immuno-receptor Tyrosine-based Activation Motif, ITAM); 3) a short cytoplasmic tail. Unlike TREMs, TREMLs possess an immuno-receptor domain within the intracellular tail, consisting of a tyrosine-based inhibitory motif (ITIM), which is an inhibitory member of the TREM family [3,7,8]. TREM and TREML endogenous ligands include the B7 family member protein B7-H3, anionic residues from the wall surface of GRAM-positive and -negative bacteria, as well as components expressed by astrocytoma cells [6,9]. Besides MAMPs, even Danger-Associated Molecular Patterns (DAMPs), namely non-pathogen-derived intracellular molecules that are released from damaged cells, may activate TREM proteins, leading to the inflammatory response [10].

In line with this, alterations in TREMs expression are bound to a variety of infectious, sterile inflammatory, and degenerative conditions, ranging from IBD to neurodegenerative disorders [3,11]. In detail, TREM-1 is expressed on neutrophils and monocytes/macrophages, where it similarly exerts pro-inflammatory effects in both infectious and non-infectious diseases. As shown by very recent studies, besides IBD, TREM-1 is overexpressed in neurodegenerative disorders, such as Alzheimer’s disease (AD) and ischemia/stroke, where it may contribute to disease pathophysiology [12,13,14]. At the molecular level, TREM-1 promotes an exuberant immune response and fuels the production of pro-inflammatory chemokines and cytokines. This occurs either by synergizing with the nucleotide-binding and oligomerization domains of NLRs or TLRs [15], or via activating the extracellular signal-related kinase 1/2 (ERK1/2), and the phosphoinositide 3-kinase (PIK3)/phospholipase C-gamma (PLC) pathway [5]. TREM-2 is expressed on macrophages, immature monocyte-derived dendritic cells, osteoclasts, and microglia. Contrarily to TREM-1, TREM-2 may be protective, especially in the CNS, where it promotes microglial phagocytosis. Paradoxically, in IBD, TREM-2 may produce detrimental effects similar to TREM-1, since targeting TREM-2 locally in the intestine has been shown to counteract inflammation [3]. 

Besides plasma membrane-bound TREMs, soluble forms of these receptors (sTREMs) have been described as well. Formation of sTREMs can be due to either alternative splicing of the *TREM* gene or cleavage of TREM protein domain. In any case, the release of sTREMs correlates with the severity of inflammation, and they suppress TREM signaling through neutralization of their ligands [3]. 

The present review provided an update on TREM-1 and TREM-2, bridging inflammation in intestinal and neurodegenerative disorders. While providing a general overview of the role of microbiota in gastrointestinal (GI) diseases and the microbiome-gut-brain axis as a route for spreading inflammation to the CNS, a special emphasis was dedicated to TREMs pathophysiology in relationship with intestinal dysbiosis, IBD, and neurodegeneration. Links between TREMs activation and alterations in the autophagy pathway were discussed since autophagy alterations occur as a common signature in both IBD and neurodegenerative disorders. 

## 2. Microbiota and Gastrointestinal Diseases

The gastrointestinal (GI) tract contains more than 35,000 species of commensal and pathogenic bacteria that have co-evolved with the human genome. Strict anaerobes represent the most abundant population of the intestinal microbiota, largely prevailing over facultative anaerobes and aerobes. Although more than 50 bacterial phyla have been reported within the human gut microbiota, *Bacteroidetes* and *Firmicutes* dominate over *Proteobacteria*, *Verrucomicrobia*, *Actinobacteria*, *Fusobacteria,* and *Cyanobacteria*. In humans, the major bacterial end-products are the short-chain fatty acids (SCFAs) acetate, propionate, butyrate, as well H2 and CO2, ammonia, amines, phenols, and energy, which bacteria use for growth and the maintenance of cellular function [2,16]. SCFAs are likely to mediate the chemical trafficking with intestinal endocrine cells (“microbial endocrinology”) whose alteration accounts for functional GI dysmotility. They also play a fundamental role in promoting barrier functions by increasing the expression of tight junction proteins, such as claudin. Finally, specific bacterial strains can produce and release serotonin, dopamine, and noradrenaline [16]. Apart from bacteria, the gut includes other microbial domains, such as archaeal genera (for example, *Methanobrevibacter smithii*), an extensive virome with bacteriophages, eukaryotic microorganisms with fungi and protists (*Candida*, *Malassezia*, *Saccharomyces*, and *Blastocystis*) [17].

This microbial population colonizes the gut at birth, and its number increases distally, reaching the peak in the colon, differing in composition and function based on its location, age, sex, ethnicity, and diet. The microbiota takes a pivotal role in the development of the immune system and immunomodulation processes. Under normal conditions, the GI mucosa, especially at Paneth cells level, produces antimicrobial peptides, including defensins. The interaction between the GI tract and resident microbiota is well-balanced in healthy individuals, but its alteration, with bacterial overgrowth and loss of competition leading to pathogen shift, can participate to the onset of intestinal (IBD, irritable bowel syndrome, and GI cancer) and extra-intestinal disorders (cholelithiasis, liver damage, obesity, allergy, type 1 diabetes, familial Mediterranean fever) [2,16,18].

It is hypothesized that chronic IBD may occur in genetically predisposed subjects due to a dysregulated and aberrant immune response to gut luminal constituents, including commensal bacteria that penetrate the intestinal mucosa. Although an inter-individual microbial variability exists in the feces of healthy subjects, a consistent alteration of the microbiota in amount and quality occurs in IBD patients. An important distinction considers the mucosa-associated microbial population from the fecal microbiome, which resides in the gut lumen without direct contact with the intestinal epithelium. Changes of adherent bacteria appear more involved in the development of inflammatory diseases, leading to the concept of “disease-predisposing microbiota” or “pathobionts”, that is, opportunistic bacteria derived from commensal ones within fecal microbiota. The main intestinal bacterial populations endowed with pro-inflammatory activity include *Escherichia coli*, *Enterococcus* species, and *Bacteroides* subspecies [19].

Epithelial barrier dysfunction, with tight junction disruption and microvilli alterations, causes paracellular and transcellular hyperpermeability, respectively, which precedes the onset of intestinal mucosal inflammation. In particular, it seems that increased bacterial internalization within epithelial cells occurs before the onset of tight junction damage, allowing other luminal bacteria without strain specificity to enter paracellular spaces up to the underlying lamina propria, where they cause mucosal inflammation [19].

Altered fecal microbiota composition and enrichment of mucosa-associated bacteria are also reported in patients with colorectal carcinoma or familial adenomatous polyposis. In particular, *Escherichia coli*, *Fusobacterium nucleatum*, and *Bacteroides fragilis* are recognized as protumoral pathobionts that induce oxidative DNA damage and mucus degradation [19].

Quorum sensing process is the bacterial ability to release and detect specific signaling molecules and to respond to cell population density by regulation of gene transcription. Such a process occurs also in the human gut, and several signaling acylhomoserine lactone molecules produced by Gram-negative bacteria are identified in feces from both patients with GI diseases and healthy subjects. In in vitro experiments on human colon cancer cells (HCT-8/E11), some quorum sensing peptides (Phr0662, EntF-metabolite, *Enterococcus faecium*, and EDF-analog, *Escherichia coli*) were found to promote tumor progression, angiogenesis, and metastasis. Thus, apart from inflammation, the microbiota can also play a role in intestinal tumorigenesis [2,18,20].

Microbiota dysbiosis and intestinal barrier impairment are associated with the development of several chronic inflammatory disorders and systemic diseases besides IBD and GI cancer. These include celiac disease, multiple sclerosis, rheumatoid arthritis, ankylosing spondylitis, psoriasis, type 2 diabetes, allergic diseases, cardiovascular, and neurodegenerative disorders. Recently, the occurrence of common factors involved in the pathogenesis of chronic polygenic diseases led to propose the “common ground hypothesis”, which considers microbiota dysbiosis, leaky gut, and inflammation as pivotal mechanisms operating in a wide set of disorders [19,21]. In this scenario, endogenous and exogenous factors would cause gut barrier impairment and poor immune activation, leading to selective pressure on the intestinal microbiota. Thus, an initial epithelial barrier alteration associated with a low amount of passive bacterial internalization would be the first step in inducing an altered microbial community, with the propensity to an irreversible virulence due to the conversion of opportunistic bacteria to pathobionts [19].

**Figure 1:**
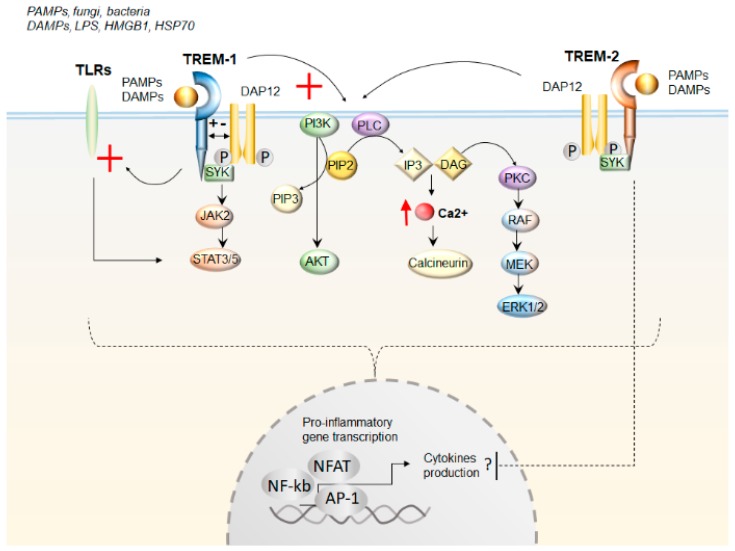
The effects of TREMs (Triggering Receptors Expressed on Myeloid cells) activation upon ligand binding. Both TREM-1 and TREM-2 are activated upon binding of either Pathogen- or Danger- Associated Molecular Patterns (PAMPs and DAMPs, respectively). TREMs activation occurs upon interaction with the 12-kDa DNAX Activating Protein (DAP12) and with the spleen tyrosine kinase (SYK), leading to subsequent stimulation (red cross) of the signaling pathways Janus Kinase/Signal Transducer and Activator of Transcription JAK/STAT, phosphatidylinositol 3-kinase (PI3K)/AKT phospholipase C (PLC), Ca2+/calcineurin, and protein kinase C (PKC)/extracellular-signal-regulated kinase (ERK1/2). TREM-1 also potentiates Toll-like receptors (TLRs) signaling (red cross). Altogether, these cascades converge in activating pro-inflammatory gene transcription through the transcription factors—a nuclear factor of activated T cells (NFAT), activator protein 1 (AP-1), and nuclear factor-κB (NF-κB). Contrarily to TREM-1, TREM-2 may inhibit cytokine production, likely by negatively regulating TLRs response, though this is dependent on disease specificity and level of inflammation. PIP2 Phosphatidylinositol 4,5-bisphosphate; PIP3 Phosphatidylinositol (3,4,5)-trisphosphate; IP3 inositol triphosphate; DAG diacylglycerol. Lipopolysaccharide (LPS); High Mobility Group Box 1(HMGB1); Heat Shkock Protein 70; rapidly accelerated fibrosarcoma/mitogen-activated protein kinase kinase (RAF/MEK)

Remarkably, altered expression of TREMs in the gut has been associated with abnormal inflammatory reactions and tissue destruction typically observed in patients affected by IBD [22]. This is in line with evidence indicating that lipopolysaccharide (LPS), Gram-positive and -negative bacteria, and fungi can up-regulate the expression of TREMs (Figure 1). However, as reported by a clinical study on UC patients, the increase in sTREM-1 is not to be necessarily related to bacterial infections, since patients with infectious colitis were excluded from the study and blood cultures were found to be sterile [23]. As support, TREMs are implicated even in sterile inflammatory-related conditions, including neurodegenerative disorders, such as AD, Parkinson’s disease (PD), Multiple Sclerosis (MS), and stroke, among others [12,13,14,24,25,26,27,28,29]. It is remarkable that a vast body of evidence is emerging, indicating that bowel inflammation is bound to the pathogenesis of CNS disorders. This is best exemplified by PD, which is linked to IBD by both epidemiological and genetic evidence. As recently reviewed, a bidirectional link between IBD and PD is strongly supported by findings suggesting a role for bowel inflammation in the initiation and progression of neurodegeneration [30]. Therefore, in the next sections, we discussed evidence centered on the role of TREM proteins in GI disorders with a focus on IBD, before moving to the role of TREMs as a bridge in the inflammatory process occurring in IBD and CNS disorders. 

## 3. Role of TREMs in the Pathophysiology of Gastrointestinal Disease 

### 3.1. TREM-1

Inflammation is a physiological response to noxious stimuli, including bacterial pathogens. However, an excess of constitutive immune activity can trigger abnormal inflammatory patterns, leading to tissue damage up to disease. In the healthy bowel, TREM-1 is constitutively expressed on neutrophils and monocytes. During acute inflammation, TREM-1 stimulation promotes the release of several pro-inflammatory cytokines and neutrophil degranulation, thus acting as an amplifier of the immune response. Interestingly, unlike monocytes and macrophages from secondary lymphoid tissues (splenic tissue, tonsils, and lymph nodes), a large amount of macrophages from the human intestinal lamina propria lacks TREM-1 expression. This may be due to the action of IL-10 and TGF-beta, which synergistically prevent TREM-1 up-regulation. Thus, the absence of TREM-1 on intestinal macrophages may be due to an adaptation of resident cells to a specific environment of the intestinal lamina propria. This is expected to prevent an excess of inflammation and counteracting tissue damage [22,31].

To date, the natural ligands of TREM-1 remain largely unknown. However, in vitro studies documented that bacterial and fungal stimuli activated TREM-1 in neutrophils and monocytes. This produces pro-inflammatory effects through the intracellular pathways PI3K/PLC and ERK1/2 [5,32]. Thus, TREM-1 and bacterial products evoke inflammatory responses through mutually stimulating pathways [5]. At intracellular levels, this leads to DAP-12 dependent Ca^2+^ influx and activation of Jak/STAT signaling along with transcription factors, such as ELK1, nuclear factor of activated T cells (NFAT), activator protein 1 (AP-1), and nuclear factor-κB (NF-κB). In turn, these transcribe genes that encode pro-inflammatory cytokines, chemokines, and cell-surface molecules (Figure 1). Again, TREM-1 synergizes with NLRs or TLRs in potentiating the immune response and releasing pro-inflammatory chemokines and cytokines [15]. Again, this occurs through the activation of NF-κB, which in turn, up-regulates the expression of TREM-1 through a feedback loop.

In line with this, TREM-1 and sTREM-1 have been reported in a variety of inflammatory diseases, including chronic rheumatoid disease [33], sepsis, and pneumonia [34]. In these conditions, elevated levels of TREM-1 and sTREM-1 are detected in synovial fluid, serum, and broncho-alveolar lavage fluid, respectively. TREM-1 and sTREM-1 also increase in non-microbial inflammations, such as psoriasis and vasculitis [5]. Likewise, sTREM-1 is increased in the gastric juice of patients affected by a peptic ulcer, where sTREM-1 may act as a mediator of pathogenesis [35].

Consistently, TREM-1 and sTREM-1 are considered as possible mediators in the pathogenesis of chronic IBD, as assessed in both experimental models and IBD patients [22,36]. In detail, in a mouse model of dextran sodium sulfate-induced colitis, TREM-1 up-regulation within intestinal macrophages increases the secretion of pro-inflammatory mediators. Conversely, when animals are administered a TREM-1 antagonist (LP17 peptide), either before or after colitis induction, pathological alterations and inflammatory mediators are suppressed. TREM-1 activity is tightly bound to the endoplasmic reticulum (ER) and autophagy [4]. In detail, in experimental sodium dextran sulfate-induced colitis, TREM-1 inhibition ameliorates dysbiosis and reduces the severity of colitis at clinical, endoscopic, and histological levels, which goes along with alleviation of ER stress and rescuing of the autophagy pathway. Again, in a mice model of 2,4,6-trinitrobenzene sulfonic acid-induced colitis, guggulsterone suppresses intestinal inflammation and disease activity via TREM-1 down-regulation, which leads to polarization of macrophages towards M2 phenotype, and reduction of NF-κB and AP-1 [37]. Intriguingly, these effects do not occur in IL-10-, TLR4-, and MyD88-deficient mice, suggesting that the pro-inflammatory effects of TREM-1 occur through IL-10 and TLR4 signaling pathways [37]. In summary, TREM-1 is both a trigger and a chronic inducer of intestinal inflammation perpetuating inflammatory disorders [31]. This is consistent with what observed in another model of experimental colitis (RAG2−/− mice with adoptive transfer of CD4^+^ CD25^−^ CD45RB^hi^ T cells), where both colonic TREM-1 mRNA and serum sTREM-1 levels are markedly increased. However, their up-regulation in the colon vs. serum varies upon the disease state. In particular, while TREM-1 mRNA expression in the colon correlates with disease activity, increased serum sTREM-1 levels are associated with slighter disease severity [36].

IBDs (including CD and UC) are characterized by episodes of relapse (active disease) and remission (quiescent disease), which implies an accurate disease assessment in humans. This is routinely carried out by endoscopy and non-invasive evaluation of blood biomarkers, such as C-reactive protein, erythrocyte sedimentation rate, and peripheral blood leukocyte counts, as well as fecal biomarkers, such as calprotectin and lactoferrin. Since endoscopy represents an expensive and discomforting procedure for patients, TREM-1 and sTREM-1 have been regarded as potentially reliable biomarkers in IBD which is currently under validation. In particular, sTREM-1 levels correlate with disease stage and TNF-alpha levels in UC [23]. Macrophages expressing TREM-1 are significantly increased in chronic inflammatory reactions in both UC and CD patients, and TREM-1 expression in the intestinal mucosa is proposed as a reliable marker of disease activity [22]. Similar conclusions have been drawn for sTREM-1 [38]. A combination of serum sTREM-1 levels and the clinical activity index is suggested as a non-invasive complementary method in evaluating endoscopic activity among patients with UC. However, in CD, TREM-1 levels appear inappropriate for assessing endoscopic activity [39]. Saurer et al. [36] failed to find a correlation between intestinal TREM-1 mRNA expression and serum sTREM-1 levels. TREM-1 mRNA expression is selectively up-regulated in intestinal biopsies from CD and UD patients with active disease, while serum sTREM-1 is elevated in patients with both active and quiescent disease. Even in patients with active disease, the increased expression of TREM-1 mRNA does not associate with peak levels of serum sTREM-1 [36].

TREM assessment may also predict therapy efficacy in IBD. For example, serum TREM-1 down-regulation joined with a high amount of mucosal plasma cells and macrophages in colonic samples may predict non-responsiveness to anti-TNF therapy in IBD patients [40], though this approach needs to be further validated [41,42].

Considering the widely accepted connection between bowel inflammation and cancer, the pro-inflammatory activity of TREM-1 might have a role also in tumorigenesis. As previously outlined, since TREM-1 overexpression in the gut occurs only in pathological conditions, the involvement of this receptor in cancer onset is just secondary to inflammation, with resistance to cell death and induction of angiogenesis. As support, in two murine models of dextran sodium sulfate-induced colitis, and colitis-associated azoxymethane-induced tumorigenesis, administration of the TREM-1 antagonist LP17 produces anti-inflammatory effects in the colon and decreases intestinal epithelial proliferation [43]. Similar results were obtained in a recent study suggesting that TREM-1 ablation protects from colorectal cancer in an experimental model of inflammation-driven tumorigenesis [44]. In detail, compared to the tumor-free colonic mucosa, TREM-1 expression is increased in both murine and human colorectal tumors, specifically within tumor-infiltrating neutrophils. TREM1-expressing colon tumors feature an overexpression of innate pro-inflammatory genes associated with tumorigenesis, while TREM1-deficient tumors feature an increased expression of genes related to adaptive immunity [44]. 

### 3.2. TREM-2

TREM-2 is expressed on macrophages, immature monocyte-derived dendritic cells, osteoclasts, and microglia, but not on granulocytes or monocytes. Unlike TREM-1, human TREM-2 is not constitutively expressed. Nevertheless, its expression can be induced in human dendritic cells, which are grown from blood monocytes cultured in GM-CSF and IL-4. TREM-2 stimulation in these cells up-regulates CCR7, a chemokine receptor for Chemokine (C-C motif) ligand 19 CCL19 (EBI-1 ligand chemokine, macrophage-inflammatory protein 3 beta), as well as CCL21 (secondary lymphoid tissue chemokine), which plays an important role in the migration of dendritic cells to lymph nodes [3]. Thus, TREM-2 may have a role in chronic inflammation, and it may stimulate the production of constitutive rather than inflammatory chemokines and cytokines. TREM-2 activation by several bacterial and yeast compounds promotes phagocytosis [5,45]. TREM-2-deficient patients develop degenerative brain disease and bone cysts, resembling the clinical phenotype, which occurs in patients lacking DAP12.

In TREM-2 family, two closely related molecules have been identified, TREM-2A and TREM-2B. These two receptor subtypes bind to both Gram-positive and -negative bacteria, as well as to human astrocytoma but hemopoietic cell lines [3]. Anionic carbohydrates inhibit such a binding, suggesting that TREM-2 recognize pathogens via charged carbohydrates expressed on their surface. In this respect, the TREM-2 profile is similar to other PPRs, including the scavenger receptors, complement receptor 3, CD14, and many TLRs. However, in spite of the capability of TREM-2 to bind to bacteria, neither humans nor mice deficient in TREM-2 show increased susceptibility to bacterial infection, suggesting the occurrence of other mechanisms in host defense [3].

In a mouse model of colonic mucosal wound healing, TREM-2 on infiltrating macrophages is required for efficient mucosal repair [46]. TREM-2 knockout KO mice possess slow and incomplete wound healing along with reduced epithelial proliferation and increased macrophages infiltrate [46]. However, the opposite results are reported when assessing the role of TREM-2 in IBD pathogenesis. In IBD patients, TREM-2 expression is markedly up-regulated within dendritic cells infiltrating the lamina propria of the inflamed mucosa [45]. This is reminiscent of what observed in two murine models of experimental colitis, namely dextran sodium sulfate and 2,4,6-trinitrobenzene sulfonic acid [45]. In this context, genetic ablation of TREM-2 is associated with protection against colitis, along with the reduced secretion of pro-inflammatory cytokines and matrix metalloproteinases expression. Furthermore, TREM-2-deficient dendritic cells exhibit a reduced ability to release pro-inflammatory cytokines, eliminate bacteria and activate T cells in response to bacteria-associated antigens. This suggests that in the gut microenvironment, TREM-2 might be an amplifier of inflammation, thus a potential target for the treatment of IBD. In particular, TREM-2 expression may amplify dysfunctional NOD signaling-mediated inflammation [45]. These findings were reproduced by McVicar et al. [47], who showed that TREM-2 deletion ameliorated dextran sulfate sodium-induced colitis and colitis-associated cancer, which is induced by azoxymethane-dextran sulfate sodium. In detail, in animals with colitis-associated cancer, TREM-2 is overexpressed in monocytic myeloid-derived suppressor cells and tumor-associated macrophages. On the other hand, TREM-2-KO animals have less severe colitis, reduced cytokine production in the colon, along with a reduced number of mitotic epithelial cells and advanced carcinomas [47]. Thus, similar to TREM-1, TREM-2 may foster the progression of colitis-associated cancer by controlling epithelial proliferation during colonic injury and inflammation.

## 4. The Context of Microbiota-Gut-Brain Axis and Neurodegenerative Diseases

There is bidirectional communication between the intestinal microbiota and the CNS through multiple pathways: endocrine (release of hormone-like compounds), nervous (parasympathetic pathway and spinal cord), immune (cytokines), and metabolic (release of SCFAs). Compounds released by intestinal microbiota may either act locally in the enteric nervous system, or reach the brain through the blood, circumventricular organs, or the vagus nerve, forming the so-called microbiota-gut-brain axis [48,49]. Again, the mesenteric lymphatic vessels play a role in immune cells and metabolite trafficking, such as the induction of regulatory cells, which are directly responsible for suppressing autoreactive T cells that infiltrate into the CNS in pathological conditions [50]. Abnormalities in the microbiome may influence the balance between effector and regulatory T cells, which can be critical in the development of inflammatory and immune processes.

**Figure 2:**
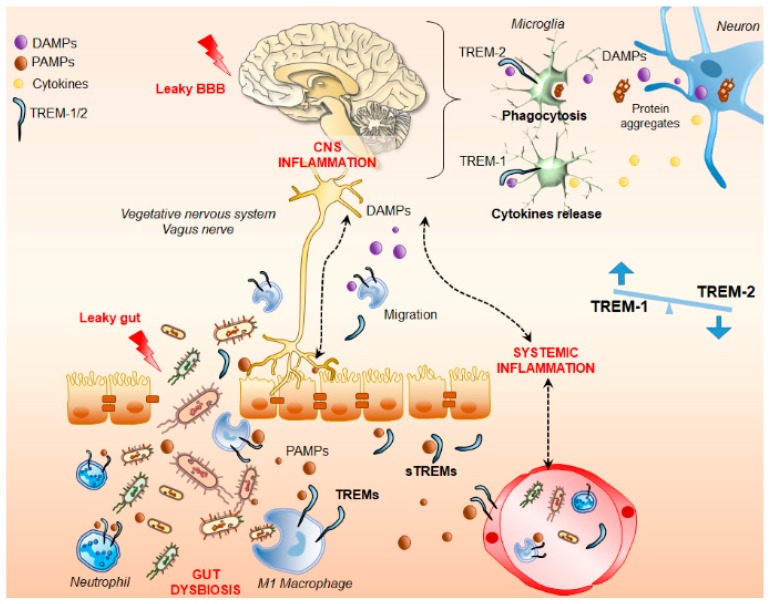
Triggering Receptors Expressed on Myeloid cells (TREMs) spreading inflammation through the gut-brain-axis. In conditions of dysbiosis, compounds released by intestinal bacteria (Pathogen-associated Molecular Patterns, PAMPs) activate TREMs on macrophages and neutrophils leading to an exuberant pro-inflammatory reaction up to damage in the gut barrier. TREM-positive activated macrophages/monocytes, along with cytokines, PAMPs, microbes, and soluble TREMs may reach the brain through the blood, circumventricular organs, or the vagus nerve through the microbiota-gut-brain axis. This leads to a systemic inflammatory response which, in turn, impairs the blood-brain barrier and promotes neuroinflammation and, ultimately, neural injury and degeneration. In this context, besides being transported within the central nervous system (CNS) through infiltrating immune cells, TREMs are also activated in microglia by Danger Associated Molecular Patterns (DAMPs) being released by damaged neurons along with protein aggregates. This leads on the one hand to microglial phagocytosis by TREM-2, and on the other hand, to the amplification of the inflammatory response exacerbating both CNS and systemic inflammation through TREM-1-dependent cytokine production.

Several lines of evidence have shown that alterations of the GIM may associate with nervous system pathologies [51,52,53]. Intestinal bacteria participate in the development and maturation of the enteric nervous system and can be responsible for immune activation through a defective gut barrier. This leads to a systemic inflammatory response which, in turn, impairs the blood-brain barrier and promotes neuroinflammation and, ultimately, neural injury and degeneration (Figure 2) [38]. In particular, while changes in certain microbial populations may cause inflammatory and immune alterations up to CNS diseases, such as multiple sclerosis, some bacteria may be protective instead [54]. The concept of “microbial endocrinology” would explain the capability of intestinal bacteria to affect homeostatic activities well beyond the gut through the release of neurochemicals acting as neurohormones in the gut-brain axis. Thus, it is not surprising that comorbidity often exists between irritable bowel syndrome with altered microbiota and CNS diseases ranging from mood disorders to neurodegeneration [2,18,52].

The autonomic nervous system provides an anatomical route for bidirectional communication in the microbiota-gut-brain axis. In particular, microbiota metabolites are sensed by roughly 80% of vagal afferent fibers, which deliver such information to the CNS for the integration of interoceptive data. This chemo- and mechanosensitive perception is indirect because vagal afferents do not cross the epithelial layer, thus sensing only the diffusion of microbial compounds or signals from enteroendocrine cells. Anti-inflammatory properties have been attributed to the parasympathetic pathway of the vagus nerve, consisting of decreased intestinal permeability and modulation of microbiota composition. Central vagal stimulation promotes anti-inflammatory responses through the activation of M2 macrophages. Accordingly, ACh release from vagal terminals inhibits the production of inflammatory molecules. In this respect, chronic stressing stimuli would minimize the cholinergic tone, favoring the onset of GI inflammatory diseases [49]. Stress-induced intestinal dysbiosis might be specifically involved in the activation of the inflammasome, leading to the onset of mood disorders. In this case, a microbiota-gut-inflammasome-brain axis is described [55].

Beyond intestinal microbiome, other specific microbial populations, such as periodontal, oral, nasal, and gastric (*Helicobacter pylori*) communities, have been incriminated for the formation and accumulation of misfolded proteins in AD and PD. Some bacteria have been described to produce amyloid [48].

Host microbiota also plays an important role in controlling maturation and function of microglia in the CNS, through the release of SCFAs. In experimental animals, the germ-free condition or a depletion of the microbiota is associated with defective microglia. Conversely, reconstitution of the intestinal microbiota or administration of SCFAs can restore microglia malformation and immaturity [56]. In the next section, we focused on the role of TREM-1 and TREM-2 in neurodegeneration.

## 5. Role of TREMs in Neurodegenerative Disorders

### 5.1. TREM-1

A role of TREM-1 in neurodegeneration has emerged only in the very last years. In 2015, the first evidence was provided indicating that variants in TREM-1 were associated with an increased burden of neuritic plaques, diffuse plaques, and Aβ density, as well as cognitive decline in patients with AD [12]. Such a variant was associated with a reduced ability of monocytes and microglia for Aβ phagocytosis, as shown in vitro [13]. TREM-1 expression levels are increased in the blood of AD patients, due to marked hypomethylation at CpG sites in the TREM1 promoter [57]. Likewise, plasma sTREM-1 is significantly increased in AD patients, where it correlates with disease progression, dementia, and total tau levels [58]. These findings added on the involvement of TREM-1 besides TREM-2 in neurodegeneration; though, to date, only a few studies have investigated the specific molecular mechanisms. In detail, Owens and colleagues [14] demonstrated that during neuroinflammation, TREM-1 and TREM-2 gene expression were regulated in an opposing manner in both murine and human microglia. In detail, administration of LPS and induction of focal transient cerebral ischemia dramatically induced TREM-1 while suppressing TREM-2 expression in microglia [14], which is reminiscent of what described in myeloid cells outside the brain, including macrophages and dendritic cells [5,59,60]. TREM-1 up-regulation and TREM-2 suppression converge in exacerbating inflammation through NF-κb activation as a common pathway. Nonetheless, a divergent regulation of TREM-1 compared with TREM-2 occurs upstream of NF-κb, depending on stimulation of specific TLRs. Contrarily to TREM-2, TREM-1 is induced only by ligands which activate TLRs via the MYD88-independent TIR-domain-containing adapter-inducing interferon-β TRIF module (i.e., TLR3 and TLR4) [14]. Such a TREM-1 specific mechanism is reminiscent of what observed in experimental IBD [37].

In experimental ischemic stroke, TREM-1 acts by activating the pro-inflammatory pathways NF-κB and inflammasome (NLRP3)/caspase-1, which occurs through interacting with spleen tyrosine kinase (SYK) [61]. TREM-1-induced SYK initiation is responsible for microglial pyroptosis, which facilitates the release of intracellular inflammatory factors. Contrariwise, TREM-1 inhibition reduces microglial M1 polarization, neutrophil recruitment, as well as chemokines and protein levels of myeloperoxidase and intracellular adhesion molecule-1 (ICAM-1). These effects are associated with protection against ischemia-induced infarction and neuronal injury, as well as potentiation of cellular proliferation and synaptic plasticity in the hippocampus [61]. 

Remarkably, novel studies unraveled that in experimental ischemia/stroke, a dramatic up-regulation of TREM-1 occurs firstly in the gut and subsequently in the CNS. This is dependent on the adrenergic nervous system, providing a link between CNS disease, systemic inflammation, and gut barrier dysfunction [62,63]. In detail, in experimental stroke, TREM-1 is up-regulated in myeloid cells within the spleen and intestine, from where it reaches the brain to magnify stroke injury [63]. Within the lamina propria, noradrenergic-dependent increases in gut permeability occur, which contribute to inducing TREM-1 on activated macrophages, further increasing epithelial permeability, facilitating bacterial translocation within the CNS and exacerbating neurological damage [62,63]. Thus, following a stroke, peripheral TREM-1 induction may amplify pro-inflammatory responses to both brain-derived and intestinal-derived immunogenic components, while targeting TREM-1-reducing gut barrier dysfunction and stroke-related cerebral injury [63]. 

TREM-1 expression is augmented in the injured spinal cord of mice models [24]. Conversely, TREM-1 ablation leads to improved locomotor function and reduced levels of peripheral nerve injury-related biomarkers and inflammation-related regulators in the murine spinal cord. In detail, TREM-1 ablation leads to a down-regulation of TLR-2, -3, -4, and -9, NF-κB, and oxidative stress markers, while enhancing anti-oxidants, such as superoxide dismutase-1 (SOD1), NAD(P)H:quinone oxidoreductase-1 (NQO-1), heme oxygenase-1 (HO-1), and nuclear factor E2-related factor 2 (Nrf2) in the injured spinal cord [24]. These results are reproduced in vitro following LPS administration, where a predominant role for HO-1 emerged. HO-1 suppression occludes the reduction in inflammation, oxidative stress, and glial cells activation, which is produced by TREM-1 ablation [24]. 

### 5.2. TREM-2 

Mutations in *TREM-2* are associated with Nasu–Hakola disease, a rare autosomal recessive pathology with presenile frontal-type dementia, systemic bone cysts, and neurodegenerative brain alterations characterized by demyelination and axonal loss [25,26]. TREM-2 alterations, mainly due to *TREM-2* mutations causing a loss-of-function, are also related to a variety of neurodegenerative diseases, including AD, PD, MS, Frontotemporal Dementia (FTD), and Amyotrophic Lateral Sclerosis (ALS) [25,26,27,28,29]. TREM-2 promotes microglial phagocytosis, which is seminal to clear apoptotic neurons and potentially harmful cell-debris, including lipidated Aβ [29,64,65,66,67]. TREM-2 also modulates TLR-mediated inflammation. Stimulating TREM-2 deficient macrophages with TLR agonists increases the levels of pro-inflammatory cytokines TNFα and IL-6 compared with TREM-2-expressing macrophages [59,68]. Furthermore, Aβ may also directly bind TREM-2 and activate TREM-2 signaling pathway, though it is still unknown how this affects inflammatory responses in microglia [69,70]. 

TREM-2 is considered as a biomarker reflecting a microglial response to neuronal injury in patients affected by AD [71,72]. Higher levels of TREM-2 due to promoter hypermethylation occur in the brains of AD patients compared with healthy subjects [73]. Intriguingly, peripheral TREM-2 mRNA levels, which are also increased in AD patients, correlate with cognitive deficits and hippocampal atrophy [74]. In AD patients, sTREM2 levels increase in the cerebrospinal fluid (CSF) and correlate with the levels of tau in CSF [75], indicating sTREM2 as a biomarker for AD. It is possible that sTREM-2 acts as a decoy receptor that inhibits full-length membrane-bound TREM-2 from binding to its ligands, though its physiological function remains elusive. In experimental models of AD, TREM-2 deficiency leads to reduced microglial clustering around Aβ plaques, suggesting that TREM-2 is required for plaque-associated microglial responses [29,70,76]. In AD mice models, a role of TREM-2 was recently identified in the context of a subtype of disease-associated microglia (DAM), which localizes in the proximity to Aβ plaques and holds the potential to restrict neurodegeneration. While initial DAM activation—that includes up-regulation of Protein Tyrosine Kinase Binding Protein (Tyrobp), Apolipoprotein E (Apoe) and down-regulation of microglia checkpoint genes—is TREM-2-independent, TREM-2 is required for the full activation of the DAM program, including phagocytic and lipid metabolism activity [77]. This is reminiscent of what occurs in mice with experimental autoimmune encephalomyelitis (EAE), where TREM-2 is highly expressed on microglial cells and macrophages, while its blockade exacerbates the inflammatory process [78,79]. The levels of sTREM-2 are also increased in the cerebrospinal fluid of MS patients [27]. 

Nonetheless, such a consensual view on the protective role of TREM-2 in CNS disorders is challenged by yet controversial results obtained in experimental age-related neurodegeneration, as well as stroke, ALS, and PD. For instance, microglial TREM-2 contributes to age-related microglial changes, phagocytic oxidative burst, and neuronal loss with possible detrimental effects during physiological aging [80]. The 24-month old TREM-2-KO mice showed a decreased age-related neuronal loss in the substantia nigra and the hippocampus compared with wild-type littermates, indicating TREM-2 as a possible contributor in age-related neurodegeneration [80]. Similar results were reported by Sieber et al. [81], who showed that the loss of TREM-2 led to an attenuation of the inflammatory response, including a reduced microglial activation in experimental stroke. On the other hand, Xu et al. [82] showed that overexpression of microglial TREM-2 in experimental ischemic stroke occurred as a neuroprotective response since TREM-2 inhibition exacerbates neuroinflammation, behavioral deficits, and cerebral infarct volume [82]. 

In 1-methyl-4-phenyl-1,2,3,6-tetrahydropyridine (MPTP) murine models of PD, TREM-2 deficiency results in reduced microglial activation and decreased expression of pro-inflammatory cytokines, albeit not affecting neuronal fate [83]. Conversely, in another study on MPTP mice models, overexpression of TREM-2 attenuates pro-inflammatory responses of microglia and protects dopaminergic neurons from MPTP-induced damage [84]. In an ALS murine model of SOD1^G93A^ mutation, TREM-2 deficiency suppresses alterations in the expression profile of microglia, suggesting that TREM-2 may switch microglia from homeostatic to an ALS-associated phenotype [85]. This is in line with what recently reported by Götzl et al. [86], showing that loss of TREM-2 enhances the expression of genes associated with a homeostatic microglial state in vivo, contrarily to the neurodegenerative microglial phenotype, which derives from the ablation of progranulin (*GRN*), an additional gene involved in neuroinflammation occurring in AD and FTD. Despite leading to opposite microglial activation states and functional phenotypes, the loss of TREM-2 and GRN results in reduced glucose metabolism in the brain, suggesting that opposite microglial phenotypes may also lead to similar brain dysfunctions [86].

Thus, TREM-2 dysregulation may contribute to the pathogenesis of neurodegenerative disorders by acting as a double-edged sword, depending on disease specificity, stage, and the role of activated microglia in different CNS disorders [87].

## 6. Autophagy as an Emerging Pathway Related to TREMs Pathophysiology

Autophagy plays a key role in keeping cell homeostasis through degradation of dysfunctional organelles and proteins. Genome-wide association studies have identified a role for numerous autophagy genes in IBD pathophysiology, especially CD. Numerous studies using in vitro and in vivo models, as well as human clinical studies, indicate that autophagy is pivotal for intestinal homeostasis maintenance, gut ecology regulation, appropriate intestinal immune responses, and anti-microbial protection [88,89]. Dysfunctional autophagy leads to disrupted intestinal epithelial function, gut dysbiosis, defective anti-microbial peptide secretion by Paneth cells, ER stress response, and aberrant immune responses [88]. Defective autophagy pathway and/or alterations in autophagy-related genes occur in various neurodegenerative disorders, both in neurons and microglia [90,91,92,93,94,95,96,97,98,99]. In fact, besides operating in neurons to guarantee proteostasis and synaptic plasticity, autophagy operates in microglia, where it affects both constitutive and adaptive immune functions, such as phagocytosis, inflammation, and antigen presentation [98]. 

Recently, TREMs pathophysiology has been linked to the autophagy pathway. Down-regulation of the autophagy-related gene *BECLIN-1* disrupts endocytic recycling of phagocytic receptors, including TREM-2 [100]. These alterations contribute to the onset of neuroinflammatory- and autoimmunity-related neurodegeneration, such as ischemia, stroke, AD, PD, HD, and MS [98,101]. In detail, alterations of autophagy have been related with TREM-2 deficiency in human AD brain and mouse AD model [71,100]. TREM2-deficient bone marrow-derived macrophages exhibit a defective anabolic state, which is associated with reduced levels of the mammalian target of rapamycin (mTOR), the master regulator of autophagy [102]. Thus, TREM-2 maintains microglia at high metabolic states through enhanced activation of the mTOR pathway. In fact, increased autophagy is detected in TREM2-deficient microglia and in AD patients carrying TREM-2 variants, suggesting that microglia attempts to compensate the mTOR defects with autophagy as a survival mechanism [102]. In fact, microglia lacking TREM-2 contain more autophagy-like vesicles as shown by electron microscopy. Again, the ratio of lipidated Light-Chain protein 3 LC3II to non-lipidated LC3I is markedly higher in microglia from AD mice lacking TREM-2 compared with controls [102]. 

On the other hand, compelling evidence indicates that TREM-1 may exert pro-inflammatory activity by impairing the autophagy pathway. In fact, in experimental sodium dextran sulfate-induced colitis, TREM-1 overexpression and pro-inflammatory activity are associated with ER stress and autophagy failure [4]. Conversely, TREM1 inhibition alleviates ER stress and rescues the autophagy pathway, which ameliorates gut dysbiosis and reduces colitis severity. In addition, oxidatively modified low-density lipoprotein (ox-LDL)-treated endothelial cells (ECs) exhibit increased TREM-1-mediated pyroptosis and decreased Sirt6-induced autophagy [103]. Conversely, blockage of Sirt6-induced autophagy augments TREM-1-mediated pyroptosis, whereas Sirt6 overexpression attenuates ECs inflammation and pyroptosis following ox-LDL treatment. 

Likewise, in experimental models of LPS- and 6-hydroxydopamine (6-OHDA)-induced PD, in vitro and in vivo administration of a synthetic peptide blocker of TREM-1 confers neuroprotection via activation of autophagy and anti-inflammatory pathways. In fact, TREM-1 inhibition significantly inhibits the up-regulation of inducible nitric oxide synthase (iNOS), cyclooxygenase-2 (COX-2), and Nf-kB, while up-regulating the autophagy-related proteins LC3 and histone deacetylase-6 (HDAC-6) [104].

These results indicate that TREM-1 and TREM-2 may cooperate to finely-tune autophagy in baseline conditions, while alterations in the TREM-1/-2 ratio may contribute to amplifying inflammation in part through altering autophagy; specific molecular mechanisms and/or signaling pathways remain to be investigated.

## 7. Concluding Remarks

Microbiota dysbiosis and intestinal barrier impairment are associated with the development of several chronic inflammatory disorders, including IBD, and neurodegenerative disorders. The primary risk factor for neurodegeneration remains advancing age, though chronic bowel inflammation may play a role in CNS disease pathogenesis. In fact, the gut microbiota plays a specific role in modulating neuroinflammation and neuro-immune functions well beyond the GI tract. Recently, TREM-2 and TREM-1 emerged as key players in those inflammatory alterations affecting both the gut and the microbiota-gut-brain axis, which might be relevant in the pathogenesis and comorbidity of IBD and neurodegenerative disorders. In this scenario, a prominent role emerges for TREM-1, which is dramatically up-regulated in the gut, from where it migrates in the CNS to promote neuroinflammation.

In summary, TREMs may provide a link between CNS disease, systemic inflammation, and gut barrier dysfunction. Nonetheless, TREM-1 and TREM-2 may also have contrasting roles in controlling myeloid cell immune activity, and their relative and co-ordinated regulation appears important, yet poorly investigated. Such a dichotomy is somewhat reminiscent of what reported for COX-1 and COX-2 in inflammation [105], suggesting that the effects of selective vs. general inhibitors of TREM-1/-2 isoforms should be carefully evaluated. In keeping with possible biochemical pathways, which are affected by TREMs alterations, autophagy appears to hold center stage. Autophagy is pivotal for intestinal homeostasis, appropriate intestinal immune responses, and anti-microbial protection, as well as neuronal and microglial functions. Thus, alterations in TREMs expression, especially TREM-1, may participate in the pathophysiology and comorbidity of IBD and neurodegeneration by altering the cell-clearing systems. Beyond autophagy, it would be worth testing the effects of TREMs in relation with the proteasome and its immune-related isoform, the immune-proteasome, which acts as a sentinel in the cross-talk between the immune system and CNS [106]. In summary, dissecting the role and fine mechanisms of action of TREMs through novel experimental strategies aimed at modulating their expression in the gut and CNS may provide novel insights, and hopefully, potential therapeutic opportunities for chronic inflammatory diseases, including IBD and neurological diseases.

## Abbreviations

**Ach**	acetylcholine
**AD**	Alzheimer’s disease
**ALS**	Amyotrophic Lateral Sclerosis
**AP-1**	activator protein 1
**Aβ**	amyloid beta
**CD**	Crohn’s disease
**CNS**	central nervous system
**DAMPs**	Danger-Associated Molecular Patterns
**DAP12**	12-kDa DNAX Activating Protein
**EAE**	experimental autoimmune encephalomyelitis
**ECs**	endothelial cells
**ER**	endoplasmic reticulum
**ERK1/2**	extracellular signal-related kinase 1/2
**FTD**	Frontotemporal Dementia
**GI**	gastrointestinal
**GIM**	gastrointestinal microbiota
**HD**	Huntington’s disease
**HDAC-6**	histone deacetylase-6
**HO-1**	heme oxygenase-1
**IBD**	inflammatory bowel disease
**ICAM-1**	intracellular adhesion molecule-1
**iNOS**	inducible nitric oxide synthase
**ITAM**	Immuno-receptor Tyrosine-based Activation Motif
**ITIM**	Tyrosine-based Inhibitory Motif
**LDL**	low-density lipoprotein
**LPS**	lipopolysaccharide
**MAMPs**	Microbe-Associated Molecular Patterns
**MPTP**	1-methyl-4-phenyl-1,2,3,6-tetrahydropyridine
**MS**	Multiple Sclerosis
**mTOR**	mammalian target of rapamycin
**NFAT**	nuclear factor of activated T cells
**NF-κB**	nuclear factor-κB
**NLRP3**	inflammasome
**NLRs**	Nod-Like Receptors
**NQO-1**	NAD(P)H:quinone oxidoreductase-1
**Nrf2**	nuclear factor E2-related factor 2
**PAMPs**	Pathogen-Associated Molecular Patterns
**PD**	Parkinson’s disease
**PIK3**	Phosphoinositide 3-kinase
**PLC**	Phospholipase C
**PRRs**	Pattern Recognition Receptors
**SCFAs**	short-chain fatty acids
**SOD1**	superoxide dismutase-1
**STREMs**	soluble Triggering Receptors Expressed on Myeloid cells
**SYK**	spleen tyrosine kinase
**TLRs**	Toll-Like Receptors
**TREML**	TREM-like transcripts
**TREMs**	Triggering Receptors Expressed on Myeloid cells
**UC**	ulcerative colitis
**6-OHDA**	6-hydroxydopamine
